# Supplementary material for: A sequential one-pot approach for rapid and convenient characterization of putative restriction-modification systems
Source: mSystems. 2023 Oct 16;8(6):e00817-23. doi: 10.1128/msystems.00817-23 (PMC10734518; doi:10.1128/msystems.00817-23)
Supplement: Supplemental material — Fig. S1 to S10; Tables S1 to S3. [file msystems.00817-23-s0001.pdf]

Supporting information

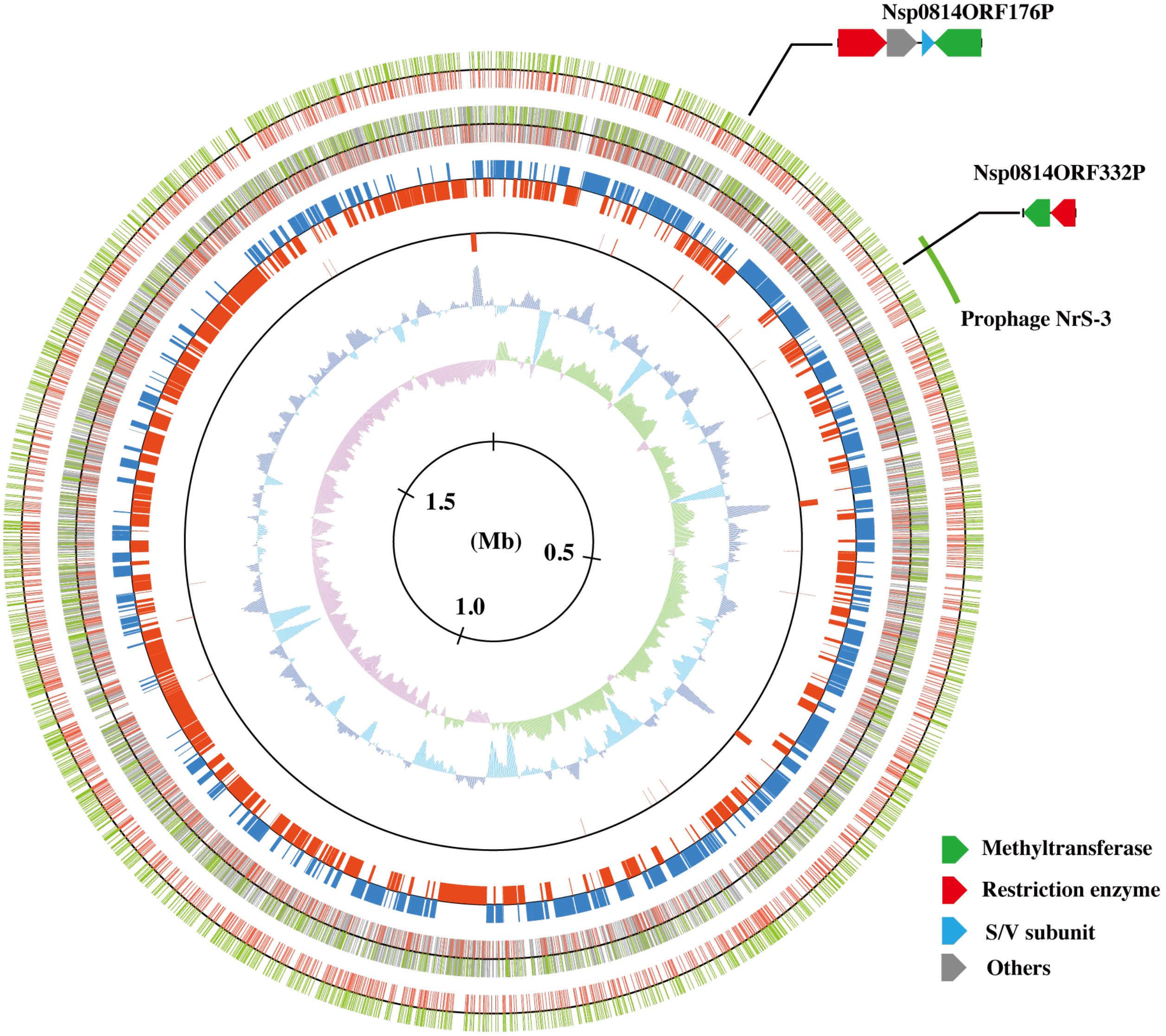

Fig. S1. Genome organization and DNA methylation landscape of the lysogenic bacterium *Nitratiruptor* sp. YY08-14. From the inside, the first and second circles showed the GC skew (values either greater or less than zero are indicated in purple or green, respectively) and the G+C percent content (values either greater or smaller than the average percentage in the overall chromosome are shown in sky blue or blue, respectively), respectively. The third circle showed the locations of rRNA and tRNA genes. The fourth circle showed CDS genes (those on the forward and reverse strands are colored in blue and red, respectively). The fifth and sixth circles showed the locations of the motifs GCNGC and RGATCY, respectively. The methylated motifs were indicated either in green on the forward or red on the reverse strand. The non-methylated motifs were colored in gray. In the outermost layer, the integrated prophage NrS-3 and three

putative R-M enzymes (the putative R-M system of NrS-3 and another putative methyltransferase in the host side) of interest in this study were also depicted. The circular representation of the genome was drawn with a customized TCL/TK script.

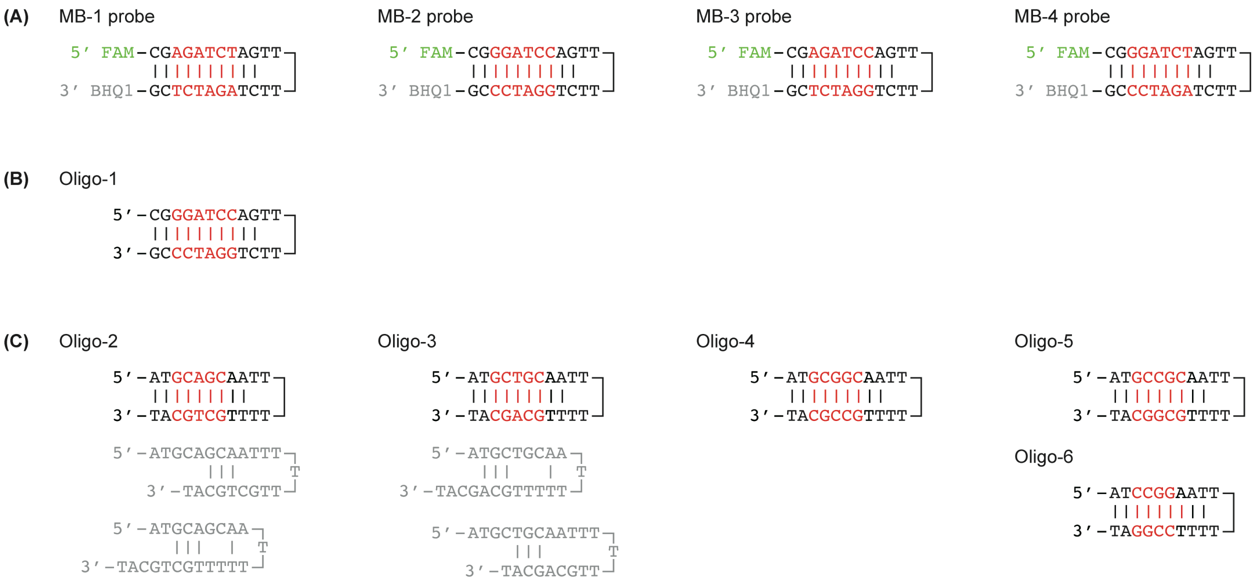

Fig. S2. DNA oligos used for R-M enzyme assays in this study. (A) The hairpin structure of the molecular beacon (MB) probes. The red-colored nucleobases constitute the predicted recognition motif RGATCY. (B) The hairpin-shaped DNA oligo substrate designed for the putative MTase NRS3_07. This oligo has the same sequences as the MB-2 probe but without terminal modifications. (C) The hairpin-shaped DNA oligo substrates designed for the putative MTase C0176. The red-colored nucleobases in Oligo 2~5 constitute the predicted recognition motif GCNGC. Oligo-6 was designed to contain an irrelevant recognition sequence CCGG (arbitrarily one of the top hit motifs predicted by the BLASTP search) and was used as a negative control substrate. Oligo 7 (Molecular Biology Insights) also detected two other possible hairpin shapes (grey-colored) for oligo-2 and oligo-3, respectively. Because the predicted T_m (from 12.6 °C to 1.2 °C) were much lower than the assay temperature (37 °C), their hairpin structures would not be present in the assay solution.

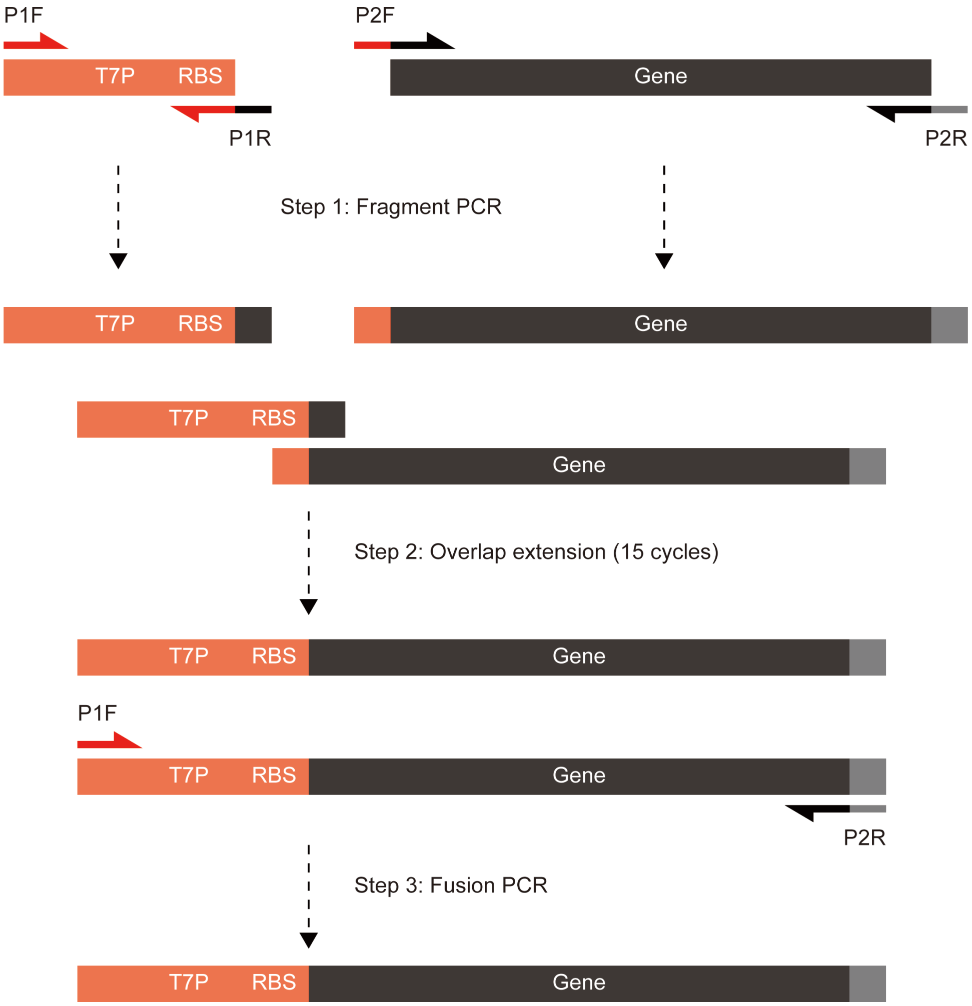

Fig. S3. Overlap extension PCR for the preparation of linear template DNA compatible with the PURE system. The distinct advantage over the suppliers' standard protocol based on an extraordinarily long (76~85 bp) forward primer is the relatively short primers (P1F, P1R, P2F, P2R) with similar T_m (Table S3), which allows specific and efficient PCR reactions. The 5' end (light grey-colored) of the P2R primer contains at least ten arbitrary nucleotides downstream of the stop codon of the target gene. The annealing temperature of the step-1 PCR is calculated based upon the 3' end of the gene-specific portion of the primer, not the entire primer. Similarly, the annealing temperature of the step-2 overlap extension is calculated based on the annealed region of two fragments. T7P: T7 promoter; RBS: ribosome binding site.

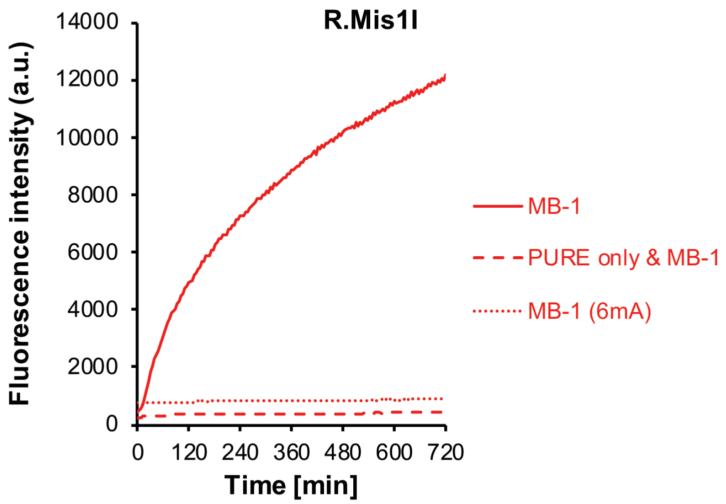

Fig. S4. The MB probe-cleavage assay for another putative REase from a giant virus. The MB-1 probe was arbitrarily selected and used for the restriction activity assay. The corresponding N6-methyladenine-modified (at the GATC motif) MB probe was also used to determine its DNA methylation sensitivity.

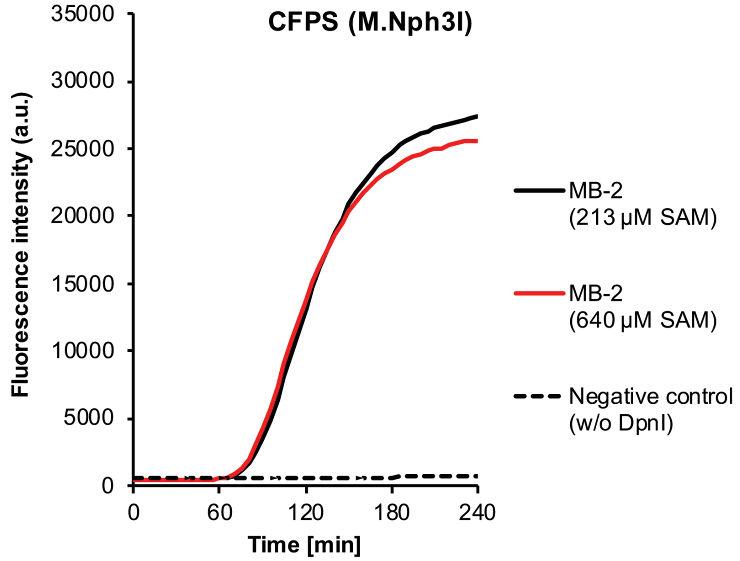

Fig. S5. CFPS of M.Nph3I coupled with the probe methylation reaction and methyl-directed restriction assay. No DpnI was added to the negative control reaction. The result also revealed that the PURE system was little affected by high concentrations of SAM, even over 0.6 mM.

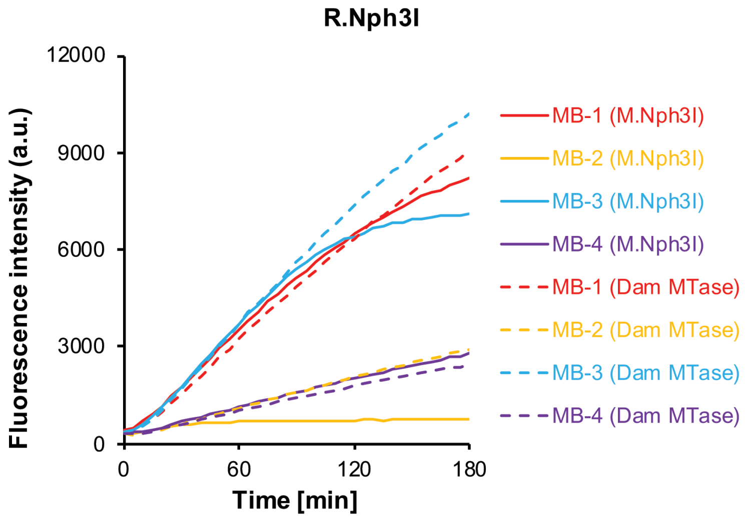

Fig. S6. Restriction assay for methylated MB probes prepared with MTase (M.Nph3I or *E. coli* Dam MTase). The DNA methylation reaction was first carried out with excess methyl donor SAM for an extended time. The crude DNA solution was then subjected to the restriction reaction with R.Nph3I.

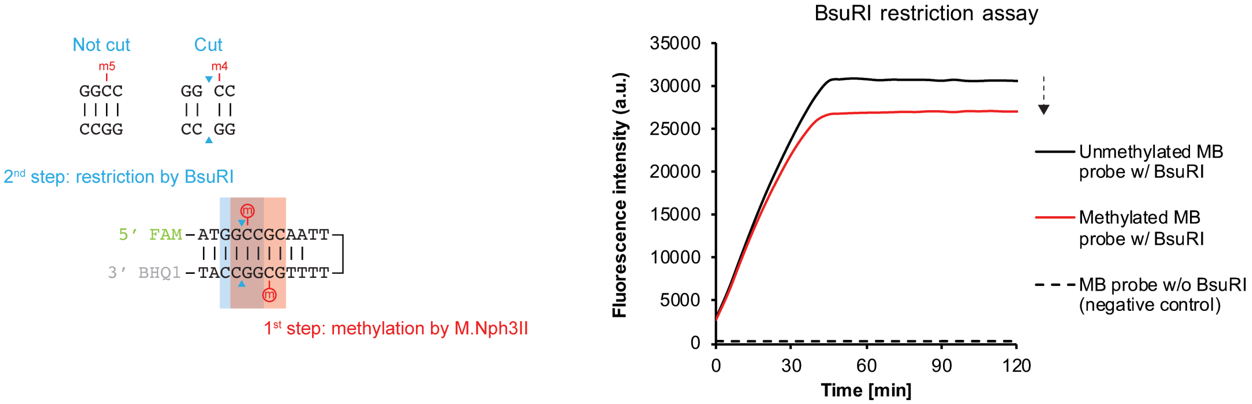

Fig. S7. Discrimination of different methylation types of the cytosine. An MB probe was designed to contain two motifs being partially overlapped with each other. One motif (GCCGC) was recognized and methylated by M.Nph3II. Then, the methylated probe, whose solution might still contain unmethylated DNA molecules, was subjected to restriction cleavage by BsuRI, an REase sensitive to (or blocked by) 5mC methylation at the specified cytosine of its recognition motif GGCC (refer to http://rebase.neb.com/cgi-bin/msget?BsuRI). The restriction reaction would not be blocked if the cytosine was methylated to 4mC. The experimental result showed a

12% decrease in the cleavage efficiency toward the methylated probe solution compared with the unmethylated one, suggesting that M.Nph3II is the 5mC type.

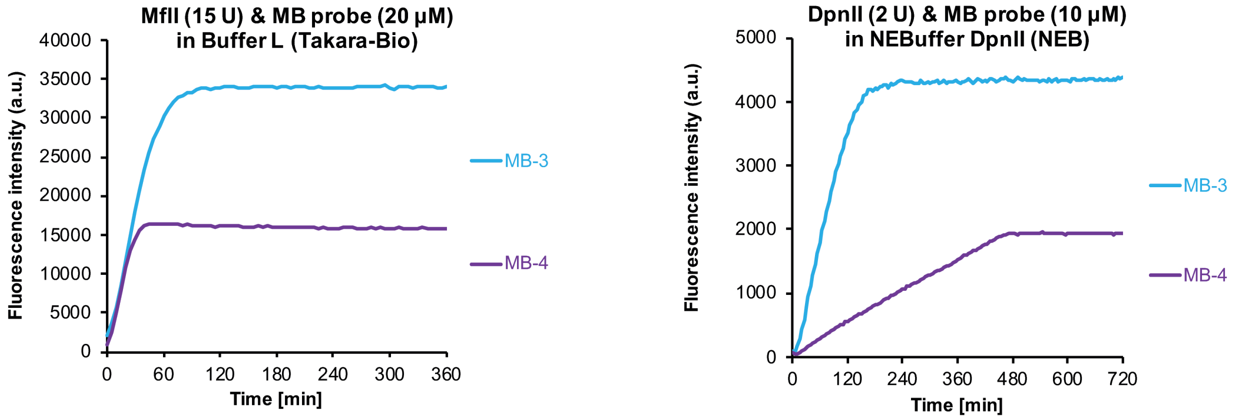

Fig. S8. MB probe cleavage reaction by MflI (recognition motif: RGATCY) or DpnII (recognition motif: GATC) in the respective optimal reaction buffers. The probe concentration (20 or 10 μM) was measured based on OD_{260} and was given by the oligo manufacturer upon delivery. To digest the DNA as fully as possible, excessive enzyme or extended reaction time was applied to the reaction. Since both charts showed different fluorescence intensities between MB-3 and MB-4 at the plateau and the degree of the difference was similar, the actual concentration was probably different from the given one.

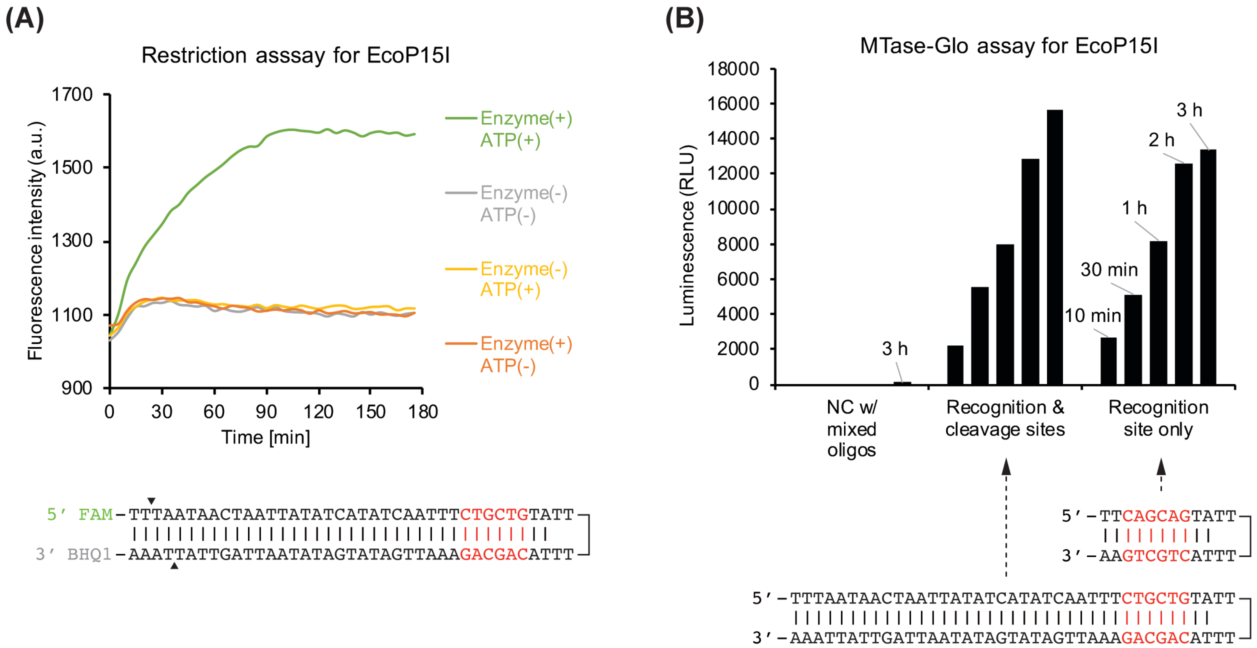

Fig. S9. Characterization of a type III R-M enzyme EcoP15I. (A) The restriction assay using an MB probe that contains a single recognition site (red-colored bases) and a cleavage site (black arrows). Oligo 7 confirmed the sole hairpin structure formation of this probe. The result confirmed the ATP-dependent REase activity of EcoP15I. 5 mM ATP, 20 µM MB probe, and 5 U EcoP15I were applied in a total 15 µL reaction solution. (B) The methylation assay using two kinds of DNA substrates. One substrate has the same DNA sequence as the above MB probe but without terminal modifications; the other one has only the recognition site (red-colored bases) and no cleavage site. The methylation reaction was carried out for 10 µM DNA in the presence of 5 U EcoP15I and 50 µM SAM (without ATP) for 10 min, 30 min, 1 h, 2 h, or 3 h. The result confirmed the MTase activity of EcoP15I toward both substrates, proving that the MTase activity is independent of the cleavage site. A negative control experiment (buffer only, without EcoP15I) was also carried out with the mixture of those two DNA substrates and SAM for 3 h.

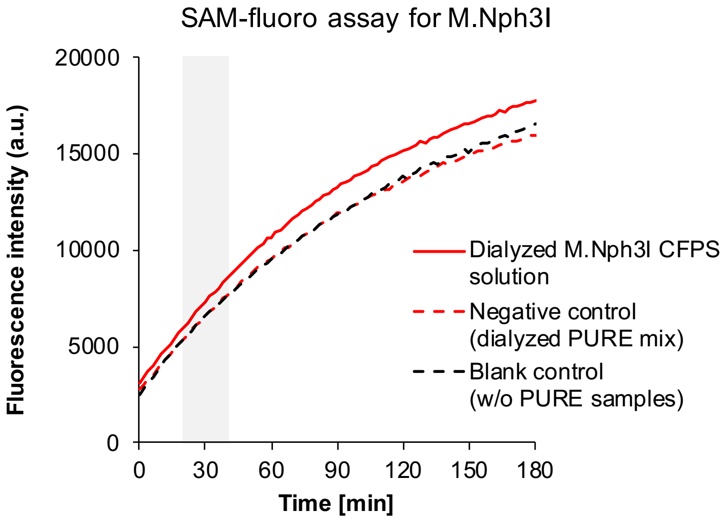

Fig. S10. A fluorogenic assay (SAM-fluoro assay) for the detection of MTase activity of M.Nph3I. The assay procedure just followed the protocol offered by the kit's supplier. The same dialyzed samples used in Fig. 3E were also used herein. The dialysis process was necessary to remove reducing agents from the PURE solution that may inhibit the assay. The grey-shaded area was subjected to linear regression to calculate the initial reaction rate representing the enzyme activity. The negative control (dashed red line) showed an identical fluorescence profile to the blank control (dashed black line), where no PURE components were added. Most notably, both control samples showed high background fluorescence. The fluorescence contributed by the methyl transfer reaction (solid red line) was only about 14% above the background.

Table S1. Methylation motif detection from reanalysis of the PacBio sequencing data of *Nitratiruptor* sp. YY08-14 genome. In brief, CCS reads containing at least three full-pass subreads on each polymerase read and with >99% average base-call accuracy were retained as HiFi reads using the standard PacBio secondary analysis tools on bioconda with default settings. For epigenomic analysis, the HiFi reads were converted to add conventional kinetic information using the ccs-kinetics-bystrandify script included in the PacBio software package. The HiFi reads were mapped to *Nitratiruptor* sp. YY08-14 genome (composed of a chromosome [NZ_AP023061.1] and a plasmid [NZ_AP023062.1]) using pbmm2, an official wrapper software for minimap2[1], for the calculation of interpulse duration ratios. Modification detection and motif prediction were performed using ipdSummary and MotifMaker (with a '--minScore 30' setting), respectively, included in the SMRT Link package (v10.2). Motifs with ambiguous sequences

probably generated by misdetection were manually curated. Specifically, a 5mC-type motif G(^{5m}C)DGC was detected, while the internal 'D' was likely due to incomplete detection of the motif. Considering the experimental result (Fig. 3F), the motif most likely represents G(^{5m}C)NGC.

Predicted methylated motif	Modification type	Number of modified sites	Number of motif sequences in genome	Modification ratio (%)
RG**A**TCY	6mA	3870	3870	100.0
G**C**NGC	5mC	4189	9744	43.0
TTG**A**TCAA	6mA	741	802	92.4

R=A/G, Y=C/T, N=A/C/G/T

Bold characters indicate modification sites.

Table S2. Primers used for In-Fusion cloning of the NRS3_08 gene.

PCR product	Template	Primer	Sequence
Linearized vector	pET-3a plasmid	Forward	TAACAAAGCCCGAAAGGAAGCTGAG
		Reverse	CATATGTATATCTCCTTCTTAAAGTTAAACAAAATTATTTCTAGAGGGA
Insert	NrS-3 genomic DNA	Forward	GGAGATATACATATGGGGATTGATCTACTGCCATCT
		Reverse	TTTCGGGCTTTGTTAACTTTCTATATACAATTTATCTGTAATCCCAAAAACGAGAATTGG

Table S3. Primers used for overlap extension PCR in this study.

Gene	PCR for Fragment 1			PCR for Fragment 2			Overlap extension	Fusion PCR
	Primer 1_Forward (P1F)	Primer 1_Reverse (P1R)	T_m difference (°C)*1	Primer 2_Forward (P2F)	Primer 2_Reverse (P2R)	T_m difference (°C)	Overlapped region (bp)	T_m difference (°C)*2
mel_015		tttcctCCATATGTATATCTCCTTCTTAAAGTTAAAC	3.0	aaggagatatacatATGGAGGAAATCATTAGCAACT	ggattagttattcaTTATTCCGATGACAGGTAGATTTC	0.4	24	3.4
NRS3_07	CATACCCACGCCGAAACAAG	gaacgacatCCATATGTATATCTCCTTCTTAAAGTTAAACAAAA	0.6	aggagatatacatATGGATGTCGTTCAGGATTT	ggattagttattcaTTAAGTTACTGATATGATATTTTTTGAATTATATTTG	0.6	26	1.5
C0176		tatgcttttCCATATGTATATCTCCTTCTTAAAGTTAAAC	3.0	aaggagatatacatATGGAAAAGCATATATATAAAATCTT	ggattagttattcaTCAGATTAGGGACTTAAATTTTTTTG	3.1	27	2.1

*1 The T_m value of each primer was calculated by a designated T_m Calculator (https://www.thermofisher.com/jp/en/home/brands/thermo-scientific/molecular-biology/molecular-biology-learning-center/molecular-biology-resource-library/thermo-scientific-web-tools/tm-calculator.html). Only the complementary region (without the 5' overhang), whose nucleotides are written in uppercase letters, was used in the T_m calculation.

*2 The T_m difference of primers used in the final fusion PCR was based on P1F and P2R (full-length) primers.

REFERENCES

1. Li H. 2018. Minimap2: pairwise alignment for nucleotide sequences. Bioinformatics 34:3094-3100.
